# Identification of *Pantoea ananatis* strain BCA19 as a potential biological control agent against *Erwinia amylovora*

**DOI:** 10.3389/fmicb.2024.1493430

**Published:** 2024-11-21

**Authors:** Jueun Lee, Won-Kwon Jung, S. M. Ahsan, Hee-Young Jung, Hyong Woo Choi

**Affiliations:** ^1^Department of Plant Medicals, Andong National University, Andong, Republic of Korea; ^2^Gyeongsangbuk-do Agricultural Research & Extension Services, Daegu, Republic of Korea; ^3^Department of Plant Medicine, Kyungpook National University, Daegu, Republic of Korea

**Keywords:** fire blight, *Erwinia amylovora*, biological control agent, *Pantoea ananatis*, indole

## Abstract

In this study, we aimed to screen potential antagonistic microorganisms against *Erwinia amylovora*, the causal agent of fire blight. From 127 unknown bacterial isolates tested, 2 bacterial strains (BCA3 and BCA19) were identified to show distinct antagonistic activity against *E. amylovora* in agar plate assay. Phylogenetic analysis of the 16s rRNA sequence identified both BCA3 and BCA19 as *Pantoea ananatis*. Among these BCA19 showed 13.9% stronger antagonistic activity than BCA3. Thus we further characterized antagonistic activity of BCA19. Culture filtrates (*CF*) of BCA19 significantly inhibited the swimming and swarming motility of *E. amylovora*. Ethyl acetate and n-butanol extracts of *CF* of BCA19 exhibited antibacterial activity in disk diffusion assay. Furthermore, gas chromatography–mass spectrometry analysis of ethyl acetate and n-butanol extracts of *CF* of BCA19 identified antibacterial compounds, including indole and hexahydropyrrolo[1,2-a]pyrazine-1,4-dione. Importantly, indole inhibited growth of *E. amylovora* with IC_50_ value of 0.109 ± 0.02 mg/mL (~930.4 μM). Whole genome sequence analysis of BCA 19 revealed gene clusters related with siderphore, andrimid, arylpolyene and carotenoid-type terpene production. This study indicates that BCA19 can be used as a potential biological control agent against *Erwinia amylovora*.

## Introduction

*Erwinia amylovora* is a Gram-negative, motile, and rod-shaped bacterium belonging to the family Enterobacteriaceae. Annual losses of apple and pear production due to this pathogen are significant in many countries (Aćimović et al., 2015; Dagher et al., 2020). In Korea, apples and pears rank as the top fruit crops and play a crucial role in domestic agricultural industry (Lee et al., 2023; Seo and Song, 2023). The presence of *E. amylovora* in Korea was officially documented from 2016 (Myung et al., 2016; Park et al., 2016; Song et al., 2021; Park et al., 2022; Cho et al., 2023; Lim et al., 2023). The rising incidence of this disease, particularly with antibacterial-resistant strains (which is not reported yet in Korea), is becoming a major concern for apple and pear cultivation (Kim and Yun, 2018; Ahn and Yun, 2021; Sundin and Wang, 2018; Ham et al., 2022; Ham et al., 2024). The World Health Organization (WHO) has published a priority list for antibiotic-resistant bacteria, such as Enterobacteriaceae, due to the urgent need to discover and develop new treatments. Therefore, new treatment measures are critically required to curb *E. amylovora*.

Antagonistic microorganisms enhance agricultural productivity and offer alternative ways to control the disease, which can compensate synthetic pesticides. The genus *Pantoea* represents a highly diverse group of bacteria (Gavini et al., 1989). This genus also belongs to the Enterobacteriaceae family and currently includes 20 species: *P. agglomerans*, *P*. *allii*, *P. ananatis*, *P*. *anthophila*, *P*. *beijingensis*, *P. brenneri*, *P. calida*, *P. conspicua*, *P. cypripedii*, *P. deleyi*, *P. dispersa*, *P*. *eucalyptii*, *P. eucrina*, *P*. *gavinae*, *P*. *rodasii*, *P*. *rwandensis*, *P. septica*, *P. stewartii, P. vagans*, and *P*. *wallisii* (Walterson and Stavrinides, 2015). Among these, *Pantoea ananatis*, a Gram-negative bacterium from the Erwiniaceae family, is a well-known phytopathogen isolated from various ecological niches and plant hosts. However, this bacterium also offers various beneficial characteristics, such as promoting the growth of host plants and increasing crop yield. Additionally, some non-pathogenic strains isolated from *P. ananatis* show promise for the microbial production of many useful substances. *P. ananatis* has been demonstrated to be an effective host for producing secondary metabolites and amino acids. It is anticipated to serve as a platform for microbial production of bioactive substances, aromatic compounds, and other high-value plant-derived substances. Furthermore, several studies have highlighted the potential of *Pantoea* spp. to induce systemic resistance and provide protection against pests and pathogenic microorganisms in cultivated plants. Strains of the *Pantoea* species merit consideration as agents for pest and phytopathogen control. Additionally, some of these strains possess biotechnological potential for therapeutic uses, such as immunomodulators. The biocontrol capabilities of *Pantoea* species largely depend on their ability to produce a diverse array of natural metabolites (Duchateau et al., 2024; Usuda et al., 2022; Lorenzi et al., 2022).

This study aims to identify and isolate antagonistic microorganisms against *E. amylovora*. From the screening, we isolated and identified *P. ananatis* strains BCA3 and BCA19 as potential antagonistic microorganisms against *E. amylovora*. BCA3 and BCA19 showed direct growth inhibition activity in agar plate assay. Culture filtrate of BCA19 significantly inhibited swimming and swarming motilities of *E. amylovora*. Gas chromatography–mass spectrometry (GC–MS) analysis of hexane and ethyl acetate extracts of culture filtrate of BCA19 revealed possible mechanisms of action of its antagonistic activity. Together, we have identified *P. ananatis* strains BCA3 and BCA19 as potential biological control agents against fire blight pathogen.

## Materials and methods

### Screening of antagonistic bacteria by agar plate assay

The selection of antibacterial microorganisms against *Erwinia amylovora* was performed via agar plate assay as previously described (Koo et al., 2023). The 127 unknown microorganisms were sourced from the Gyeongsangbuk-do Agricultural Research & Extension Service, those of which are isolated from various plants. Each bacterium and *E. amylovora* was cultured in 10 mL of Tryptic Soy Broth (TSB; 30 g/L) for 2 days in a shaking incubator at 28°C. To prepare the inoculum, OD_600_ of *E. amylovora* was adjusted to 0.2. Following the drying of 20 mL of Tryptic Soy Agar (TSA; 30 g broth and 15 g agar per liter) in a 9 cm petri dish, the surface was spread inoculated with 100 μL aliquots of *E. amylovora*. A 6 mm diameter paper disk was placed on the surface of *E. amylovora-*inoculated TSA media, then inoculated with 20 μL of each culture of unknown bacterium. Clear zone formed around the disk, indicating antimicrobial activity due to the diffusion of antimicrobial substances from the unknown bacteria. Microorganisms demonstrating antimicrobial activity were selected for further testing by measuring the size of the clear zone diameter over a period up to 7 days. Clear zone diameter (mm) was measured by subtracting diameter of colony of unknown bacteria from diameter of total clear zone. To compare antagonistic activity of BCA3 and BCA19, other *Pantoea* spp. were obtained from Korean Agricultural Culture Collection (KACC; Table 1) and tested in the same way as described above.

**Table 1:** List of *Pantoea* spp. obtained from KACC for antagonistic activity test against *E. amylovora*.

KACC No.	Scientific name
KACC 10055	*Pantoea agglomerans*
KACC 10253	*Pantoea agglomerans*
KACC 10254	*Pantoea agglomerans*
KACC 10525	*Pantoea agglomerans*
KACC 15275	*Pantoea agglomerans*
KACC 17663	*Pantoea stewartii*
KACC 19138	*Pantoea ananatis*

### Molecular identification

The 16S rRNA gene sequencing was performed to identify candidate bacteria, BCA3 and BCA19, which showed significant antagonistic activity against *E. amylovora*. Genomic DNA was extracted using the HiGene™ Genomic DNA Prep Kit (BIOFACT, Daejeon, Korea). PCR amplification was conducted in a 50 μL reaction mixture comprising 5 μL of 2 × Taq buffer, 5 μL of 5 × band doctor, 1 μL of dNTP mix, 0.25 μL of Taq polymerase, 32.75 μL of DW, 2 μL of DNA template, and 2 μL of primers. The 16S rRNA region was amplified using the primers 27F (5′-AGTTTGATCCTGGCTCAG-3′) and 1492R (5′-GTTACCTTGTTACGACTT-3′). The PCR protocol included an initial denaturation at 95°C for 2 min, followed by 33 cycles of denaturation at 95°C for 20 s, annealing at 55°C for 40 s, extension at 72°C for 1 min, and a final extension at 72°C for 5 min. The 16S rRNA gene sequences were analyzed by Solgent sequencing services (Solgent, Daejeon, Korea). The sequence data were compared to sequences in NCBI’s GenBank^1^ to identify the closest species. Phylogenetic trees were constructed using the maximum likelihood method in the MEGA X software (Kumar et al., 2018).

### Swimming and swarming motility assays

Swimming and swarming motilities of *E. amylovora* were examined in the presence of different concentrations of culture filtrate (*CF*) of BCA 19 as previously described (Koo et al., 2023). Briefly, *Pantoea ananatis* strain BCA19 was cultured in TSB medium for 24 h and the supernatant was isolated by centrifugation at 9,447 × *g* for 10 min, and filtered with syringe filter with pore sizes of 0.22 μm. Different concentrations of *CF* of BCA19 and LB agar medium were mixed together and poured to solidify. For Swimming and swarming motility assays 0.3 and 0.6% agar was added, respectably. This soft medium was dried for about a day to fully solidify. *E. amylovora* culture suspension grown in TSB medium (24 h, 28°C) was adjusted to an OD_600_ value of 0.05, then 2 μL of bacterial suspension was drop inoculated on the center of soft LB-agar medium. Colony diameter of *E. amylovora* was measured 48 h after incubation at 28°C.

### Antagonistic activity of ethyl acetate and n-butanol extracts from *CF* of BCA19

*CF* of BCA19 was extracted using two organic solvents, ethyl acetate and n-butanol. BCA19 was cultured overnight in 1 L of TSB medium. The culture supernatant was collected by centrifugation at 12,000 × g for 20 min. The supernatant was poured into a separatory funnel and mixed with either ethyl acetate or n-butanol in a 1:3 ratio. Unknown compounds from *CF* of BCA19 was extracted by vigorous shanking and separated by standing for overnight. Then, the dissolved substances in each layer were concentrated under reduced pressure to high concentration using a rotary vacuum evaporator. Each extract was weighed and diluted to different concentrations in DMSO, then used for disk diffusion assay. For disk diffusion assay, *E. amylovora*-inoculated TSA media was prepared as described above. Sterilized 6 mm paper disk was placed on top of *E. amylovora*-inoculated TSA media, then 20 μL aliquots of different concentrations of ethyl acetate and n-butanol extracts were dropped onto the disk. Clear zone diameter was measured 3 days after incubation.

### Gas chromatography–mass spectrometry (GC–MS) analysis

The extract of culture filtrate of BCA19 was analyzed using GC/MSD System (5977A Series, Agilent Technologies, Santa Clara, CA, USA) as previously described (Heo et al., 2022). The injection volume was 2 μL and the inlet temperature was set at 280°C. Helium was injected at 1 mL/min as a carrier gas. The column oven temperature was set at 80°C for 1 min, then increased to 240°C at 20°C/min, then to 260°C at 5°C/min, then to 300°C at 20°C/min for 10 min. The transfer line was 300°C, and a mass range of 40 to 450 m/z was scanned. The result was analyzed by comparing the mass spectral data with the National Institute of Standards and Technology spectral library version 11 (NIST 11 spectral library) (Oberacher et al., 2013). The most abundant compounds with a quality score higher than 90 were selected for further research.

### Antagonistic activity of indole

Indole was purchased from Sigma-Aldrich (MA, USA), and dissolved in DMSO. Disk diffusion assay was performed as described above. DMSO was used as a solvent for indole due to its ability to dissolve a wide range of compounds with no growth inhibitory effect on *E. amylovora* in our experimental conditions. 20 μl of 100 mg/mL indole was used, and clear zone diameter was measured 3 days after incubation. To determine IC_50_ of indole, *E. amylovora* was grown in TSB with different concentrations of indole (0.005, 0.015, 0.025, 0.05, 0.15, 0.25, 0.5, 1.5, and 2.5 mg/mL) in 96-well plates. The growth of *E. amylovora* was measured for 24 h using a microplate reader at OD_600_. The growth rate (%) of *E. amylovora* was calculated using the following formula. Growth rate (%) = (OD_600_ value in the presence of different concentrations of iondole/OD_600_ value without iondole) × 100. IC_50_ value was calculated by using the Quest Graph™ IC_50_ Calculator (AAT Bioquest, Inc.)^2^.

### Whole genome analysis

Genomic DNA (gDNA) was isolated from fresh bacterial cultures in TSB medium using the Maxwell® RSC Pure Food GMO and Authentication Kit (Promega, WI, USA) according to the manufacturer’s instruction. For sequencing, the gDNA was quantified using an Epoch spectrophotometer (BIOTEK, VT, USA). The library preparation for Illumina sequencing was performed using the NEBNext® Ultra™ II FS DNA Library Prep (NEB, MA, USA) and NEBNext Multiplex Oligos, Dual index 3 (NEB, MA, USA). The library was quantified with a Qubit® 3.0 fluorometer (Invitrogen, MA, USA). Sequencing was performed on an Illumina Novaseq 6,000 (Illumina, CA, USA) using in the CJ Bioscience (Seoul, Korea).

Illumina sequencing data is quality controlled with Trimmomatic-0.36 and PhiX sequences are removed by BBMap 38.32. The data that completed preprocessing was assembled with SPAdes 3.15.3 (Algorithmic Biology Lab, St. Petersburg Academic University of the Russian Academy of Sciences). Contamination of the genome of BCA19 was checked by comparing the 16S rRNA gene fragments (contamination was not observed). Gene-finding and functional annotation pipeline of whole genome assembles used in EzBioCloud genome database. Protein-coding sequences (CDSs) were predicted by Prodigal 2.6.2 (Hyatt et al., 2010). Genes coding for tRNA were searched using tRNAscan-SE 1.3.1 (Schattner et al., 2005). The rRNA and other non-coding RNAs were searched by a covariance model search with Rfam 12.0 database (Nawrocki and Eddy, 2013). The CDSs were classified into groups based on their roles, with reference to orthologous groups (EggNOG 4.5)^3^ (Powell et al., 2014). EGGNOG/COG showed the CDS of the genome categorized by COG category and displayed as a bar chart. Clusters of orthologous gene (COG) database was devised as a method to allow phylogenetic classification of proteins from microbial whole genomes (Tatusov et al., 1997). For more functional annotation, the predicted CDSs were compared with KEGG database (Kanehisa et al., 2014). Analysis of secondary metabolites was performed using anti-SMASH analysis (v7.0.0) using the parameter ‘relaxed’ to identify hits (Blin et al., 2023)^4^. The Orthologous average nucleotide identity (OrthoANI) was calculated by comparing homologous genes between the genomes (*Pantoea ananatis* LMG2665, *Pantoea allii* LMG24248, *Pantoea stewartii* subsp. *indologenes* LMG 2632, and *Citrobacter freundii* NBRC12681) to construct a phylogenetic tree (Lee et al., 2016).

### Statistical analysis

The data were subjected to analysis of variance (ANOVA) using R statistical soft (R Foundation for Statistical Computing, Vienna, Austria). Significant differences between treatment means were determined using the least significant difference (LSD) at *p* < 0.05. All experiments were performed a least two times. For each experiment, the data were analyzed separately. The results of one representative experiment are shown here.

## Results

### Screening of antagonistic bacteria by agar plate assay

**Figure 1:**
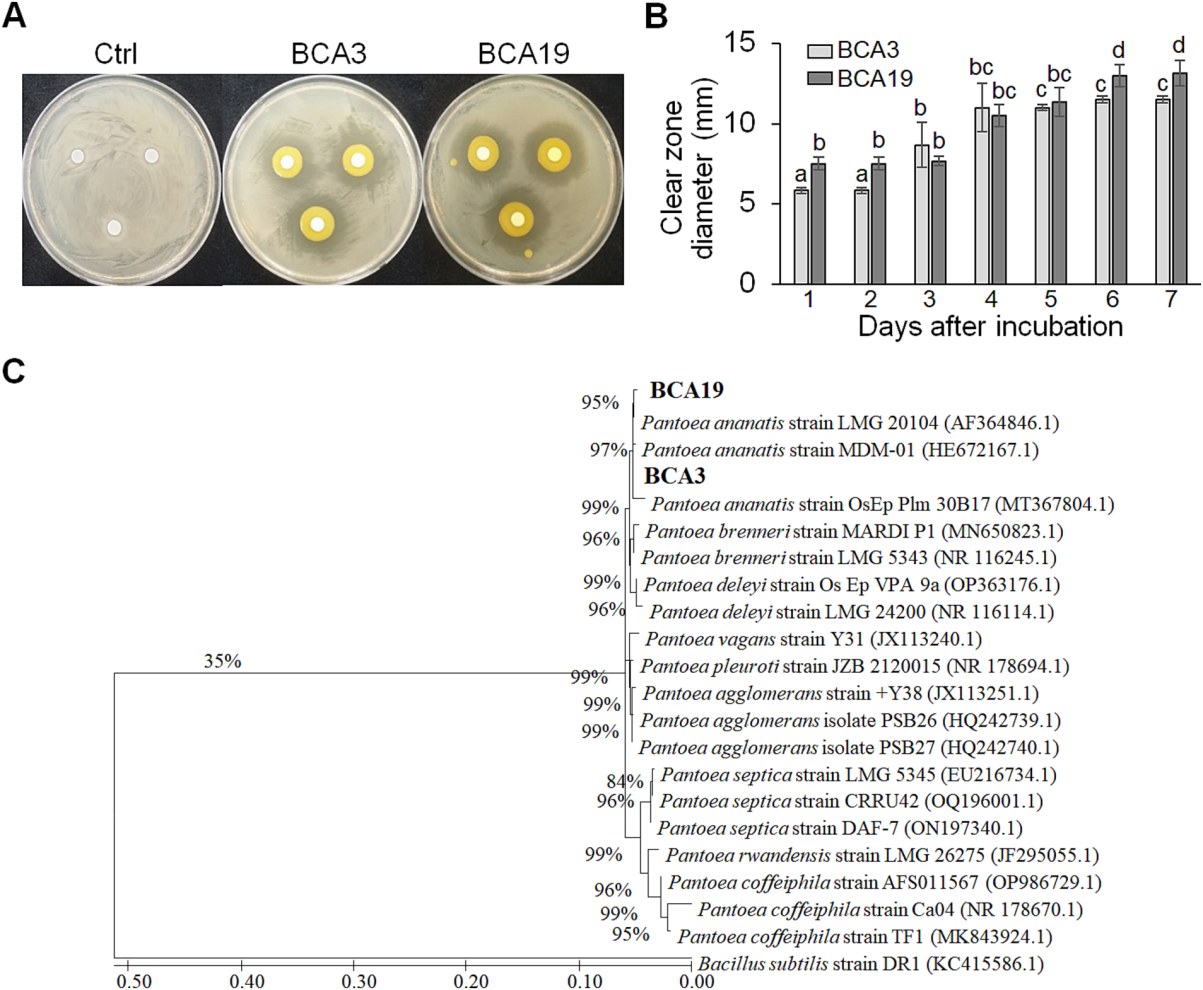
Isolation and identification of antagonistic bacteria *Pantoea ananatis* strains BCA3 and BCA19 against *E. amylovora*. **(A)** Representative picture showing antagonistic activity of BCA3 and BCA19 strains against *Erwinia amylovora*. The picture was taken 7 days after incubation. **(B)** Clear zone diameter induced by BCA3 and BCA19 against *E. amylovora*. The clear zone diameter was measured for 7 days. Experiments were performed in triplicate and repeated three times with similar results. Data are the mean ± SE (*n* = 3). **(C)** Phylogenetic tree analysis of 16s rRNA sequences of BCA3 and BCA19 with other *Pantoea* spp. The tree was generated based on MEGA X program by using maximum-likelihood method. Strain BCA3 and BCA19 were classified as the same species, *Pantoea ananatis*.

A total of unknown 127 strains were distributed by Gyeongsangbuk-do Agricultural Research & Extension Service, and used for screening. Among these, 2 strains (BCA3 and BCA19) showed distinct antagonistic activity against *E. amylovora* (Figure 1A). On day 4, diameters of the clear zone induced by BCA3 and BCA19 were 11.2 ± 4.1 and 10.5 ± 1.8 mm, respectively (Figure 1B). Interestingly, phylogenetic tree analysis using 16s rRNA revealed both BCA3 and BCA19 identified as *Pantoea ananatis* (Figure 1C). The 16s rRNA sequences of BCA3 and BCA19 showed the highest sequence similarity with that of *P. ananatis* strain OsEp_Plm_30B17 (Accession no. MT367804.1; 99.9%) and *P. ananatis* strain LMG 20104 (99.62%; Accession no. AF364846.1), respectively.

**Figure 2:**
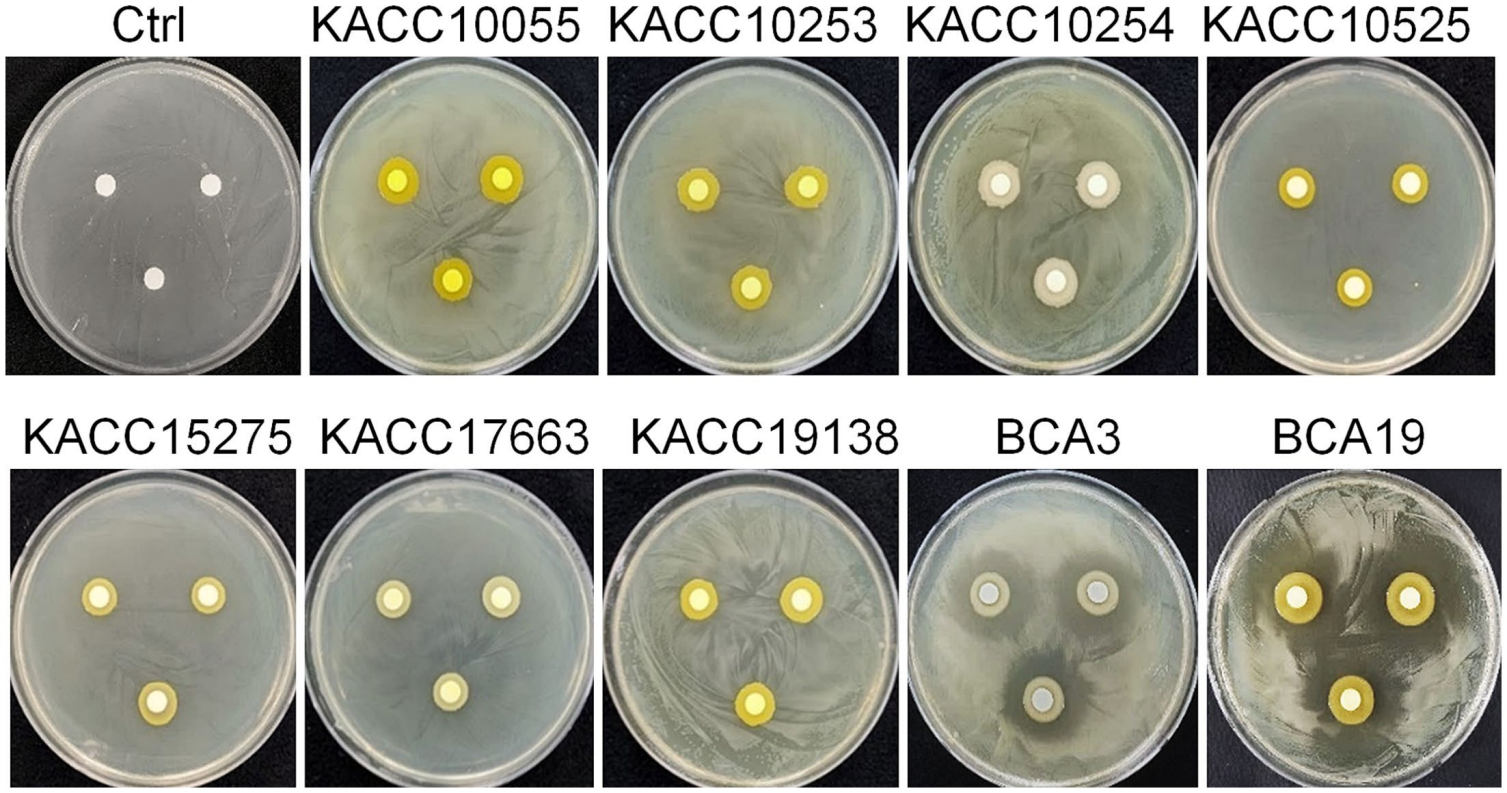
Comparison of antagonistic activity of BCA3 and BCA19 with other *Pantoea* spp. against *E. amylovora*. Other *Pantoea* spp., except *Pantoea ananatis* strains BCA3 and BCA19, failed to exhibit antagonistic activity. The picture was taken 4 days after incubation. Experiments were performed in triplicate and repeated two times with similar results.

To test whether antagonistic activity of BCA3 and BCA19 is specific or not, 7 different *Pantoea* spp. strains obtained from Korean Agricultural Culture Collection (KACC), which including 5 *P. agglomerans*, 1 *P. stewartii* and 1 *P. ananatis* (Table 1). Interestingly, none of tested *Pantoea* spp. strains, except BCA3 and BCA19, showed antagonistic activity against *E. amylovora* (Figure 2). This suggests that BCA3 and BCA19 possess highly specific antibacterial activity against the fire blight pathogen unlike other *Pantoea* spp. Notably, BCA19 showed similar but somewhat stronger antagonistic activity in agar plate assay. Thus we selected BCA19 for further analysis.

### Inhibition of swimming and swarming motility of *Erwinia amylovora* by culture filtrate of BCA19

**Figure 3:**
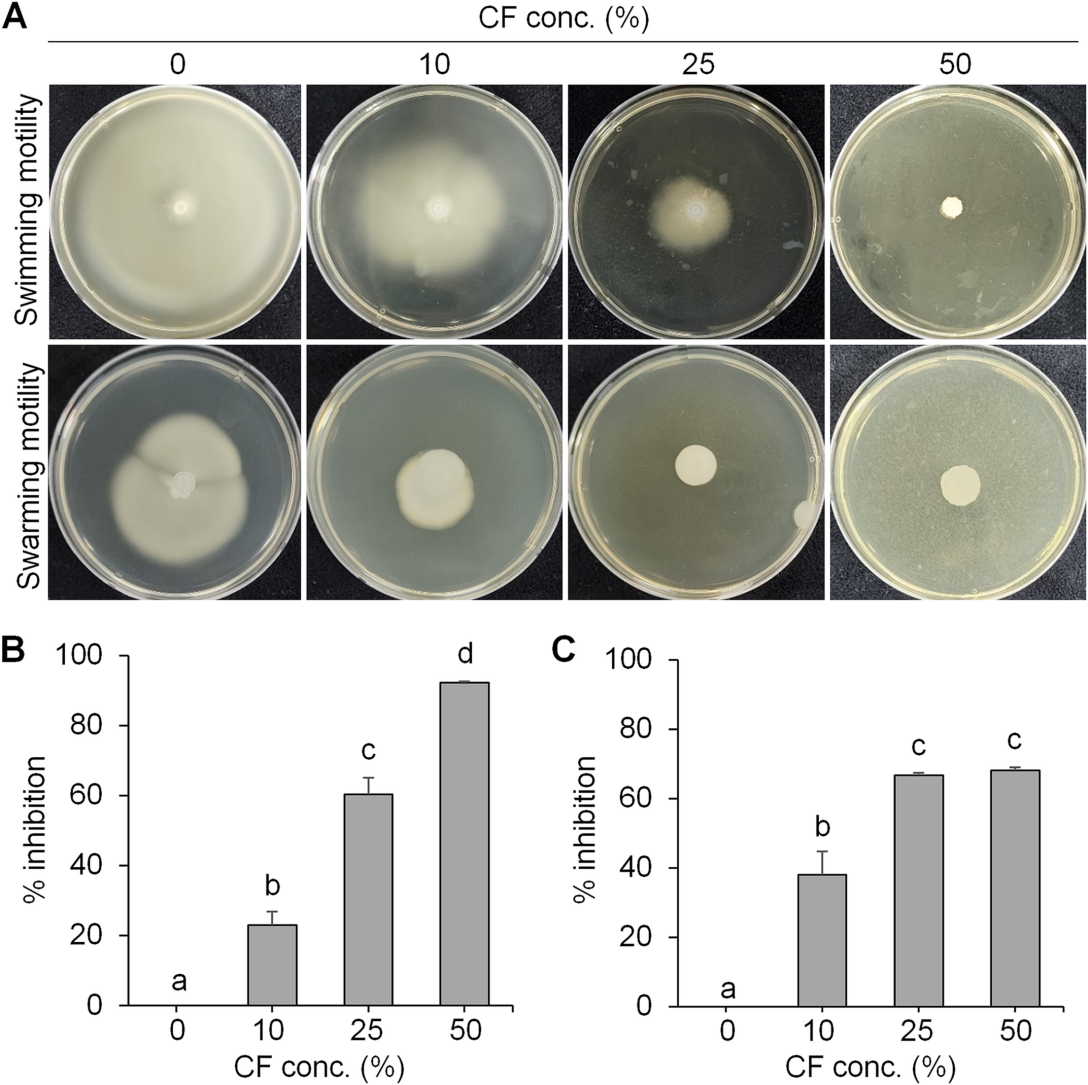
Inhibition of swimming and swarming motility of *E. amylovora* by *CF* of BCA19. **(A)** Reduced swimming and swarming motility of *E. amylovora* in the presence of different concentrations of *CF* of BCA19. **(B,C)** Inhibition rate of swimming **(B)** and swarming **(C)** motility of *E. amylovora.* Experiments were performed in triplicate and repeated three times with similar results. Data are mean ± SE (*n* = 4). Bars with the different letter indicate significant difference according to LSD test (*p* < 0.05).

To examine the antibacterial activity, swimming and swarming motility of *E. amylovora* were tested in the presence of different concentrations of culture filtrate (*CF*) of BCA19 (Figure 3A). As expected, both swimming and swarming motility of *E. amylovora* were significantly decreased by *CF* of BCA19 in a dose-dependent manner. In a LB agar media (0.3% agar) containing 10, 25 and 50% of *CF* of BCA19, swimming motility of *E. amylovora* was inhibited by 20.1, 60.4 and 92.3%, respectively (Figure 3B) In a LB agar media (0.6% agar) containing 10, 25 and 50% of *CF* of BCA19, swarming motility of *E. amylovora* was inhibited by 37.9, 66.7 and 68.2%, respectively (Figure 3C).

### Antibacterial activity of ethyl acetate and n-butanol extracts of *CF* of BCA19

**Figure 4:**
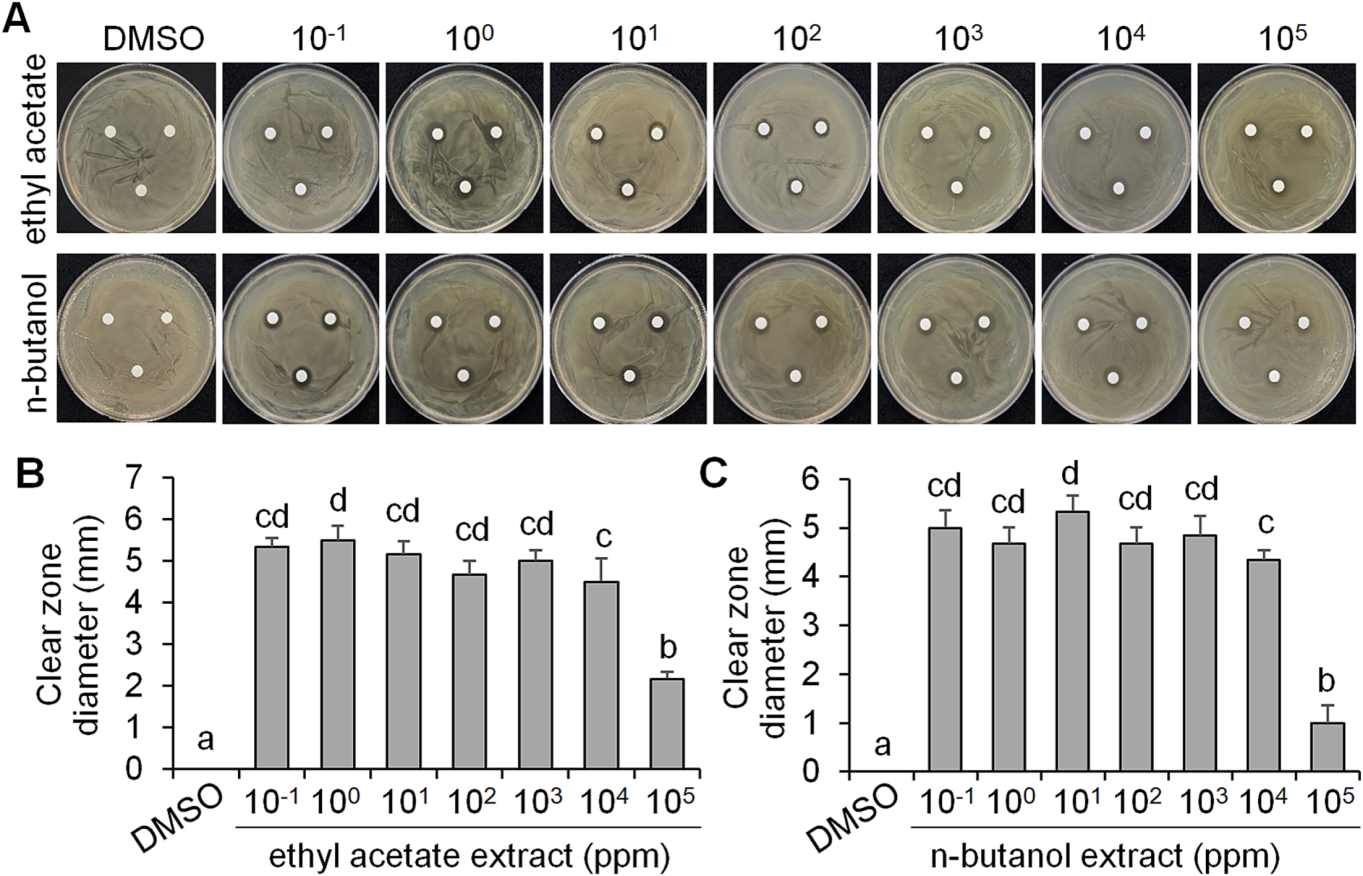
Antagonistic activity of ethyl acetate and n-butanol extracts of *CF* of *P. ananatis* strain BCA19. **(A)** Representative pictures showing the growth inhibition of *E. amylovora* in disk diffusion assay with different concentrations of ethyl acetate (top panel) and n-butanol (lower panel) extracts of *CF* of BCA19. **(B,C)** Clear zone diameter induced by ethyl acetate **(B)** and n-butanol **(C)** extracts of *CF* of BCA19. The clear zone diameter was measured on 3 days after incubation. Experiments were performed in triplicate and repeated two times with similar results. Data are the mean ± SE (*n* = 6). Bars with the different letter indicate significant difference according to LSD test (*p* < 0.05).

As *CF* of BCA19 inhibited swimming and swarming motility of *E. amylovora*, we further tested its antagonistic activity. To test whether BCA19 produce extracellular antimicrobial compounds, ethyl acetate and n-butanol extracts of *CF* of BCA19 were prepared and examined by disk diffusion assay (Figure 4A). Both ethyl acetate and n-butanol extracts showed significant antibacterial activity. Interestingly, 10^−1^ to 10^4^ mg/mL of ethyl acetate (Figure 4B) and n-butanol (Figure 4C) extracts showed similar clear zone diameter; however, 10^5^ mg/mL of ethyl acetate and n-butanol extracts showed significantly lower antibacterial activity. This suggests that optimal concentration of ethyl acetate and n-butanol extracts of *CF* of BCA19 is needed for optimal antibacterial activity.

### Gas chromatography–mass spectrometry (GC–MS) analysis of ethyl acetate and n-butanol extracts of *CF* of BCA19

To analyze secondary metabolites, ethyl acetate and n-butanol extracts of *CF* of BCA19 were analyzed by gas chromatography–mass spectrometry (GC–MS). The total ion chromatograph (TIC) corresponding to the compounds extracted with ethyl acetate and n-butanol from *CF* of BCA19 were shown in Supplementary Figure 1. In ethyl acetate and n-butanol extracts, 51 and 32 peaks were observed, respectively. From these we selected top 4 of the most abundant compounds with a quality score higher than 90 (Tables 2, 3). In ethyl acetate extract, indole was identified as the most abundant compound among different compounds identified (Table 2). In n-butanol extract, different compounds containing common hexahydropyrrolo[1,2-a]pyrazine-1,4-dione structure were abundantly identified (Table 3).

**Table 2:** List of four of the most abundant chemical compounds identified from ethyl acetate extract by GC–MS analysis.

Peak No.	Compound name	Structure	Molecular weight	Formula	Retention time (min)	Peak area (%)
5	Indole	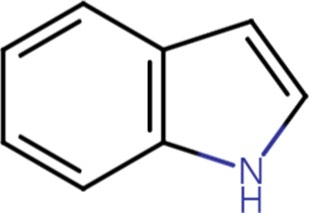	117.058	C8H7N	5.64	26.52
47	Pyrrolo[1,2-a]pyrazine-1,4-dione, hexahydro-3-(phenylmethyl)-	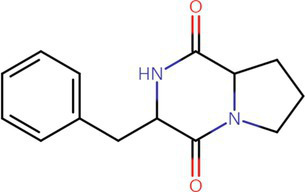	244.121	C14H16N2O2	17.11	20.54
8	Tetradecane	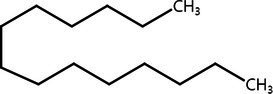	198.235	C14H30	6.41	9.01
14	Hexadecane	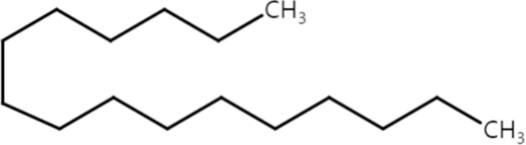	226.266		8.05	6.12

**Table 3:** List of four of the most abundant chemical compounds identified from n-butanol extract by GC–MS analysis.

Peak No.	Compound name	Structure	Molecular weight	Formula	Retention time (min)	Peak area (%)
9	Pyrrolo[1,2-a]pyrazine-1,4-dione, hexahydro-	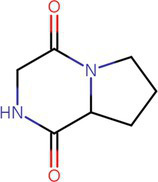	154.074	C7H10N2O2	9.601	44.46
26	Pyrrolo[1,2-a]pyrazine-1,4-dione, hexahydro-3-(phenylmethyl)-	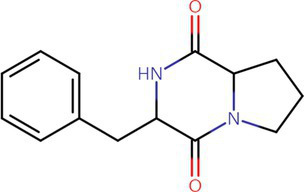	244.121	C14H16N2O2	17.082	29.99
17	Pyrrolo[1,2-a]pyrazine-1,4-dione, hexahydro-3-(2-methylpropyl)-	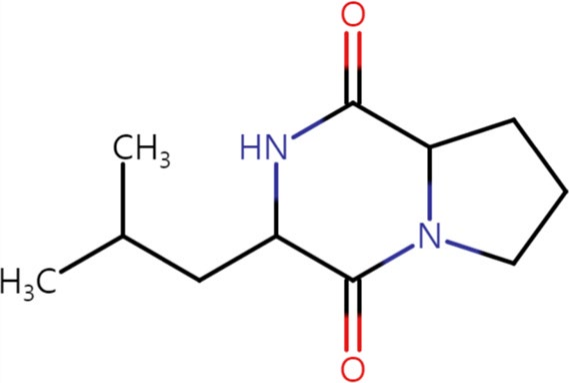	210.137	C11H18N2O2	11.301	20.89
3	Phenol, 2,4-bis (1,1-dimethylethyl)-	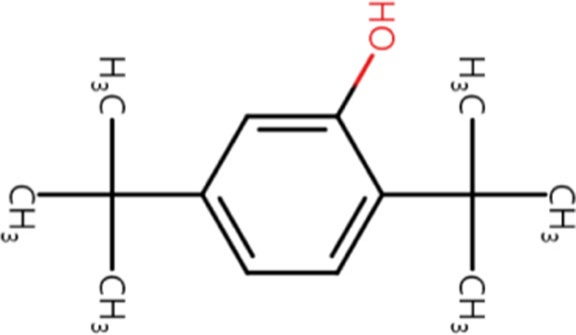	206.167	C14H22O	7.373	4.66

### Antibacterial activity of indole against *Erwinia amylovora*

**Figure 5:**
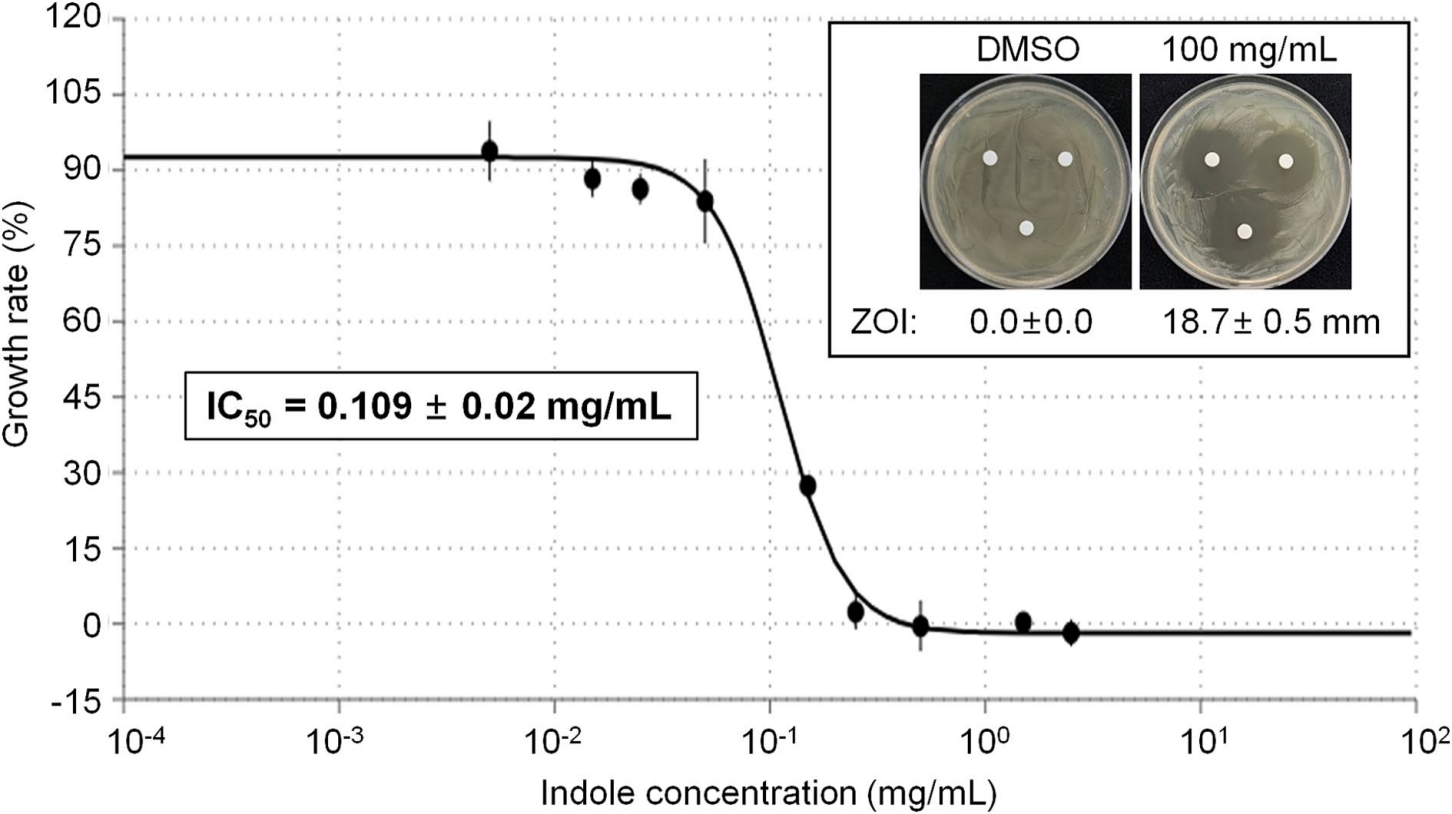
Antimicrobial activity of indole against *E. amylovora*. Representative pictures showing the growth inhibition of *E. amylovora* in disk diffusion assay with 100 mg/mL indole (Inlet). DMSO was used as vehicle. Graph showing the growth rate (%) of *E. amylovora* in the presence of different concentrations of indole. The OD_600_ value was measured in a microplate at 24 h after incubation. Experiments were performed in triplicate and repeated two times with similar results. Data are the mean ± SE (*n* = 3). Calculated IC50 was 0.109 ± 0.02 mg/mL.

Among the different compounds identified from GC–MS analysis, indole was commercially available. Thus we tested whether indole can show direct antibacterial activity against *E. amylovora*. Initial disk diffusion assay revealed that 100 mg/mL indole shows strong antibacterial activity, while DMSO (vehicle) did not show any antibacterial activity against *E. amylovora* (Figure 5, Inlet). Thus we further tested growth of *E. amylovora* in the presence of different concentrations of indole (Figure 5). As expected, indole inhibited growth of *E. amylovora* in a dose-dependent manner. The calculated IC_50_ of indole was 0.109 ± 0.02 mg/mL (~930.4 μM).

### Whole genome sequence analysis of BCA19

**Figure 6:**
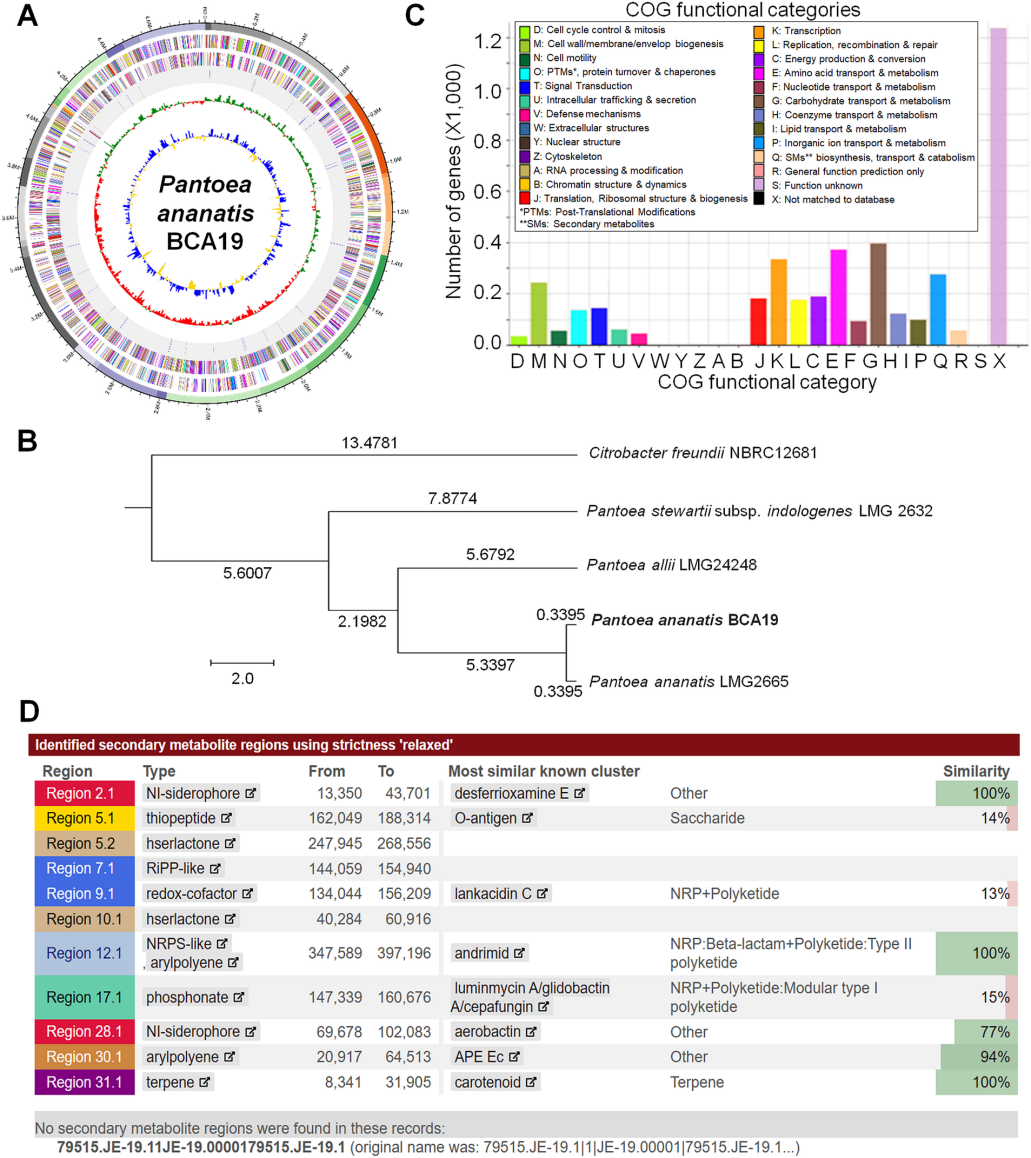
Whole genome sequence analysis of *Pantoea ananatis* BCA19. **(A)** The circular genome map of BCA19. The circular map showed CDS of DNA in the forward direction, sequence in the reverse direction, rRNA and tRNA, GC skew graph, and GC ratio graph. **(B)** Phylogenetic tree analysis of whole genome sequence of BCA19. **(C)** EggNog/COG analysis of BCA19 genome sequence. **(D)** Secondary metabolites analysis using anti-SMASH. Among the most similar known clusters, desferrioxamine E, andrimide, and carotenoids had a 100% similarity.

By sequencing the genome, we were able to determine the genomic information of BCA19 (Figure 6). The genome coverage was 423.37×, which is enough to derive high-quality draft genome assemblies. From the comparison of sequenced 16S rRNA gene fragments, contamination with other prokaryotic gDNA was not observed. The genome of BCA19 was 4,824,244 bp in size, GC content was 53.4%, and the number of CDSs was 4,394. The circular genome map represents a pseudogenome made of assembled 36 contigs (Figure 6A). Phylogenetic analysis of whole genome sequence revealed BCA19 is clustered with *Pantoea ananatis* LMG2665 (Figure 6B). All CDSs in the genomes of BCA19 were categorized by the COG category to which they belonged and represented in a bar chart (Figure 6C). The distribution of CDSs by function was confirmed. Among the various genes, 57 genes were related to secondary metabolite biosynthesis, transport, and catabolism (Figure 6C). We analyzed the genomes of BCA19 to identify biosynthetic gene clusters (BGC) of secondary metabolites using anti-SMASH (version 7.0) and identified 11 identical BGCs. Of these, three clusters had 100% similarity (Figure 6D).

## Discussion

Despite the steadily growing market share of biocontrol products relative to traditional pesticides (Prashar et al., 2013; Robin and Marchand, 2019), management of plant pathogens continues to depend heavily on chemical treatments, posing risks to human health and the environment (Barzman et al., 2015; Droby et al., 2016; Backer et al., 2018; Barratt et al., 2018; Köhl et al., 2011). The identification and selection of effective biocontrol strains through reliable methods are critically important. In this study, bacterial strains BCA3 and BCA19 of *Pantoea ananatis* were selected based on their antagonistic against the fire blight pathogen using dual culture assay. The dual culture assay is a widely utilized direct screening method for identifying biocontrol agents (BCAs) due to its simplicity and broad applicability. This assay involves co-cultivating the pathogen and the BCA on a (semi-)solid medium and evaluating the BCA’s antagonistic capacity by measuring the zone of pathogen growth inhibition (Choi and Ahsan, 2022; Choi et al., 2022; Heo et al., 2022; Koo et al., 2023; Kim et al., 2024; Woo et al., 2024). In this study, we observed a clear inhibitory effect against *E. amylovora* using a dual culture assay with our isolated *Pantoea ananatis strains*, corroborating previous findings reported for this species.

Swimming and swarming motility, facilitated by one or more polar flagella, are not only key virulence factors but also confer several advantages to bacteria (Piqué et al., 2015; Schachterle et al., 2019). These motilities enable cells to navigate leaf surfaces in search of resources, enhance nutrient absorption, compete with rival microorganisms, and evade harmful substances and stressful conditions. They also help bacteria locate and colonize optimal sites within their hosts and facilitate dispersal into the environment during transmission. While both processes are related, swimming motility refers to the movement of individual cells, in contrast to swarming motility, which involves the coordinated movement of cell clusters (Rütschlin and Böttcher, 2020; Carezzano et al., 2023). *E. amylovora* primarily uses flagella-driven swimming and swarming to move (Hossain and Tsuyumu, 2006; Zhao et al., 2009; Ha et al., 2014; Yuan et al., 2022). The diverse secondary metabolites generated from microbe–microbe interactions have been extensively studied, particularly for their impact on the swarming and swimming behaviors of bacterial populations (Rütschlin and Böttcher, 2020). In our study, the culture filtrates of *Pantoea ananatis* strain exhibited an inhibitory effect on the swimming and swarming motility of *Erwinia amylovora* in motility tests (Figure 3). In addition, ethyl acetate and n-butanol extracts of *CF* of BCA19 showed direct antagonistic activity against *E. amylovora* (Figure 4). Further GC–MS analysis revealed that *CF* of BCA19 contain nitrogen-containing heterocycles, including ‘Pyrrolo[1,2-a]pyrazine-1,4-dione, hexahydro-3-(phenylmethyl)-’, ‘Pyrrolo[1,2-a]pyrazine-1,4-dione hexahydro-’, and ‘Pyrrolo[1,2-a]pyrazine-1,4-dione, hexahydro-3-(2-methylpropyl)-’(Tables 2, 3). Nitrogen-containing heterocycles are utilized in various applications, including pharmaceuticals, organic materials, natural products, and predominantly in bioactive molecules (Kerru et al., 2020). The “Pyrrolopyrazine” contains pyrrole and heterocyclic pyrazine rings as a biologically active scaffold (Dehnavi et al., 2021). Compounds featuring this scaffold have demonstrated a range of biological activities, with antimicrobial effects being particularly significant. Moreover, numerous pyrrolopyrazine derivatives have been isolated from diverse sources such as plants, microbes, soil, and marine life. Notably, the “pyrrolo[1,2-a]pyrazine-1,4-dione” derivatives displayed significant antibacterial properties and a remarkable quorum sensing inhibition (QSI) effect against various bacteria (Singh et al., 2019; Dehnavi et al., 2021). Taken together, antagonistic activity of *CF* of BCA19 is likely due to production of nitrogen-containing heterocycles, especially pyrrolopyrazine derivatives.

Indole is a metabolite of the amino acid tryptophan, which has been shown to be involved in the regulation of a variety of bacterial physiological processes. It has been reported that indole present in *Pantoea ananatis* YJ76 not only significantly enhances the survival of YJ76 under starvation conditions (Zheng et al., 2017). In other study, indole promotes symplasmata formation, an unique multicellular aggregate structure, but inhibit biofilm formation of *Pantoea agglomerans* YS19 (Yu et al., 2016). We confirmed the antimicrobial effect of indole against *Erwinia amylovora* with IC50 of 0.109 ± 0.02 mg/mL. The antimicrobial mechanism of indole is still largely unknown. Further studies on the antimicrobial effect of indole on *E. amylovora*, including the expression of pathogenicity-related genes and swimming and swarming motility-related genes, will be needed.

Antibiotics and Secondary Metabolites Analysis Shell (AntiSMASH) results showed that BCA19 has highly conserved gene clusters (>95% homology) related with siderphore, andrimid, arylpolyene and carotenoid-type terpene production (Figure 6). Desferrioxamine E, a cyclic hydroxamic acid siderophore, is widely produced by *Streptomyces* spp., which can exhibit antimicrobial activity against certain pathogenic bacteria by limiting the pathogen’s ability to utilize ferric ions (Eto et al., 2013). Andrimid is known to blocks the carboxyl-transfer reaction of bacterial acetyl-CoA carboxylase thereby inhibiting fatty acid biosynthesis (Liu et al., 2008). It is synthesized via the non-ribosomal peptide pathway and have been reported to show broad-spectrum antibacterial activity against both Gram-positive and Gram-negative bacteria (Matilla et al., 2016). Arylpolyene (APEs) are identified in wide range of bacterial genera throughout the Proteobacteria and Bacteroidetes, which are covalently attached in the Gram-negative outer membrane thereby regulating interactions with the surrounding environment (Johnston et al., 2021). Carotenoid is a variety of natural biomolecules produced by plants, algae, yeast, fungi, and some bacteria. Carotenoid protects cells from photo-oxidative damage and have thus found important potential applications in the environment, food and agriculture, nutrition, disease prevention, and as potent antimicrobial agents (Kirti et al., 2014). Taken together, whole genome sequence analysis of BCA19 revealed potential gene clusters related with its antibacterial activity and interaction with environment. Further genetic studies will be needed to elucidate biological and/or antibacterial function of genes or gene clusters.

## Data Availability

The data presented in the study are deposited in the NCBI repository, accession numbers PQ577988 and PQ577990.

## Footnotes

^1^ http://www.ncbi.nlm.nih.gov

^2^ https://www.aatbio.com/tools/ic50-calculator

^3^ http://eggnogdb.embl.de

^4^ https://antismash.secondarymetabolites.org/
