# Supplementary material for: Identification of Pantoea ananatis strain BCA19 as a potential biological control agent against Erwinia amylovora
Source: Front Microbiol. 2024 Nov 21;15:1493430. doi: 10.3389/fmicb.2024.1493430 (PMC11617517; doi:10.3389/fmicb.2024.1493430)
Supplement: Supplementary file 1 [file Presentation_1.PPTX]

## Slide 1
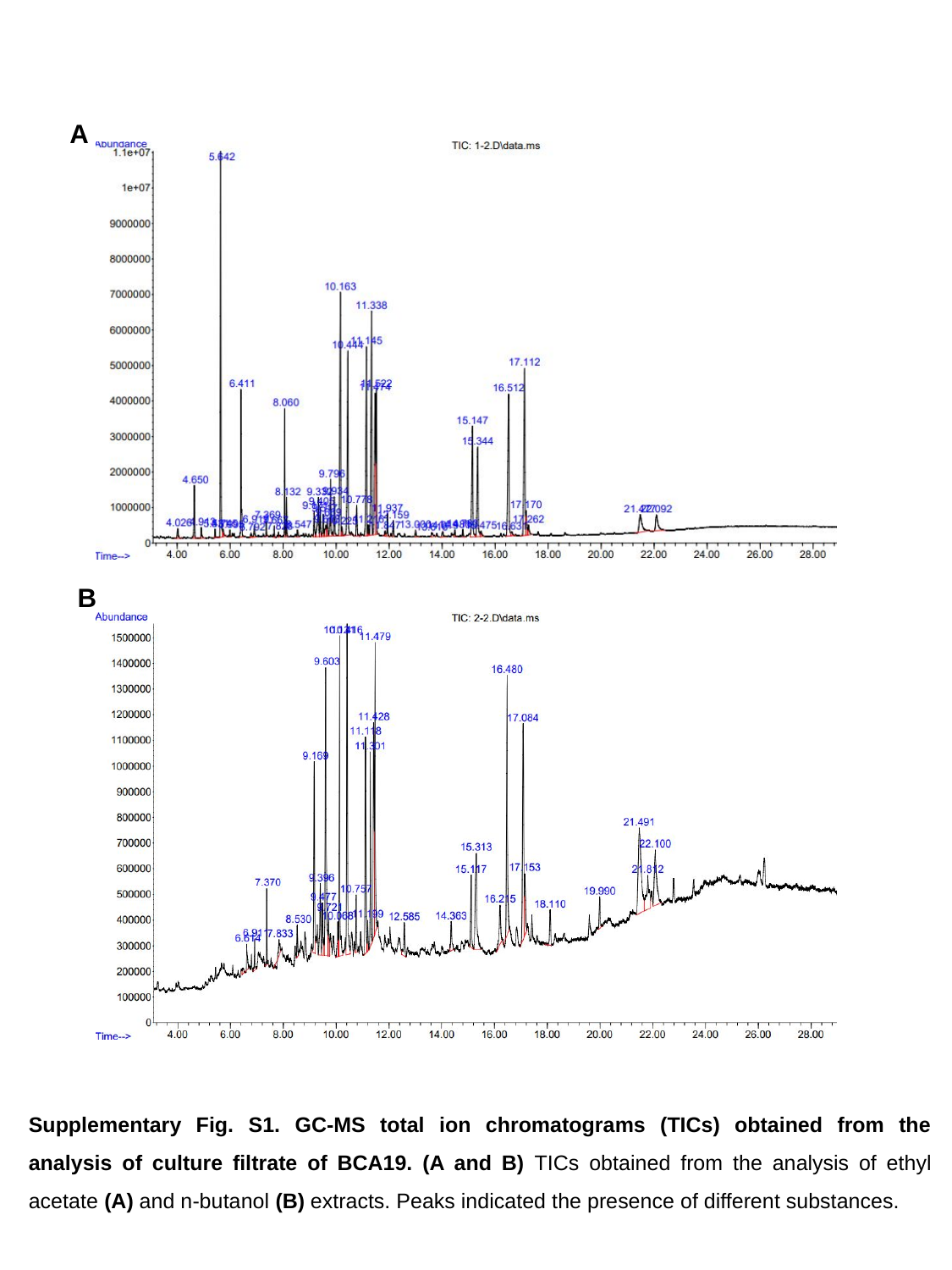

A
B
Supplementary Fig. S1. GC-MS total ion chromatograms (TICs) obtained from the analysis of culture filtrate of BCA19. (A and B) TICs obtained from the analysis of ethyl acetate (A) and n-butanol (B) extracts. Peaks indicated the presence of different substances.
